# Characterization of Extracellular Vesicles Secreted by a Clinical Isolate of *Naegleria fowleri* and Identification of Immunogenic Components within Their Protein Cargo

**DOI:** 10.3390/biology11070983

**Published:** 2022-06-29

**Authors:** Lissette Retana Moreira, María Fernanda Steller Espinoza, Natalia Chacón Camacho, Alberto Cornet-Gomez, Giovanni Sáenz-Arce, Antonio Osuna, Bruno Lomonte, Elizabeth Abrahams Sandí

**Affiliations:** 1 Departamento de Parasitología, Facultad de Microbiología, Universidad de Costa Rica, San José 11501, Costa Rica; maria.steller@ucr.ac.cr (M.F.S.E.); natalia.chaconcamacho@ucr.ac.cr (N.C.C.); elizabeth.abrahams@ucr.ac.cr (E.A.S.); 2 Centro de Investigación en Enfermedades Tropicales (CIET), Universidad de Costa Rica, San José 11501, Costa Rica; 3 Grupo de Bioquímica y Parasitología Molecular (CTS 183), Departamento de Parasitología, Campus de Fuentenueva, Instituto de Biotecnología, Universidad de Granada, 18071 Granada, Spain; acornetgomez@ugr.es (A.C.-G.); aosuna@ugr.es (A.O.); 4 Departamento de Física, Universidad Nacional, Heredia 40101, Costa Rica; gsaenz@una.ac.cr; 5 Instituto Clodomiro Picado, Facultad de Microbiología, Universidad de Costa Rica, San José 11501, Costa Rica; bruno.lomonte@ucr.ac.cr

**Keywords:** extracellular vesicles, *Naegleria fowleri*, characterization, immunogenic, proteases, proteome

## Abstract

**Simple Summary:**

Extracellular vesicles are vesicles produced by cells and organisms, capable of developing different functions. In this sense, these vesicles have been involved in communication, in the production of damage during an infection and in the modulation of different cell pathways. These vesicles have also been proposed as key players for the diagnosis and treatment of several diseases. In this study, we report for the first time the production of extracellular vesicles by *Naegleria fowleri*, the free-living amoeba that causes primary amoebic meningoencephalitis, a severe infection of the central nervous system that is fatal in more than 95% of cases. The vesicles were isolated and size, surface charge and protein content were analyzed. The report of the production of extracellular vesicles by this amoeba is relevant for different reasons: i. these vesicles could be implicated during the processes of invasion and damage to the brain; ii. they could influence the environment to trigger specific responses by cells and, iii. specific vesicles secreted by the amoeba could be employed for diagnostic purposes. Several studies to reveal the role of extracellular vesicles of this pathogen are being performed in our laboratory.

**Abstract:**

Extracellular vesicles (EVs) are small lipid vesicles released by both prokaryotic and eukaryotic cells, involved in intercellular communication, immunomodulation and pathogenesis. In this study, we performed a characterization of the EVs produced by trophozoites of a clinical isolate of the free-living amoeba *Naegleria fowleri* (*N. fowleri*). Size distribution, zeta potential, protein profile and protease activity were analyzed. Under our incubation conditions, EVs of different sizes were observed, with a predominant population ranging from 206 to 227 nm. SDS-PAGE revealed protein bands of 25 to 260 KDa. The presence of antigenic proteins was confirmed by Western blot, which evidenced strongest recognition by rat polyclonal antibodies raised against *N. fowleri* in the region close to 80 KDa and included peptidases, as revealed by zymography. Proteins in selected immunorecognized bands were further identified using nano-ESI-MS/MS. A preliminary proteomic profile of the EVs identified at least 184 proteins as part of the vesicles’ cargo. Protease activity assays, in combination with the use of inhibitors, revealed the predominance of serine proteases. The present characterization uncovers the complexity of EVs produced by *N. fowleri*, suggesting their potential relevance in the release of virulence factors involved in pathogenicity. Owing to their cargo’s diversity, further research on EVs could reveal new therapeutic targets or biomarkers for developing rapid and accurate diagnostic tools for lethal infections such as the one caused by this amoeba.

## 1. Introduction

Extracellular vesicles (EVs) are a heterogeneous group of nano to micro-sized vesicles, naturally released by both prokaryotic and eukaryotic cells. These vesicles are commonly surrounded by a phospholipid bilayer [1] and contain a bioactive cargo of proteins, metabolites, nucleic acids (DNA and RNA) and lipids. The three main subtypes of EVs include apoptotic bodies, microvesicles and exosomes, which are differentiated based upon their biogenesis, release pathways, size, content and function [2]. First considered as cell waste products or “platelet dust” [3], it has been confirmed the role of EVs in the regulation of physiological processes, emerging as key players in intercellular communication mainly through their ability to transfer biological content [4]. Furthermore, these vesicles have also been involved in pathogenesis, modulation and evasion of the immune response in pathophysiological processes. Diverse research groups have also evaluated the potential role of EVs for diagnostic (liquid biopsy) and therapeutic purposes [5,6,7].

Regarding parasites, results from different studies suggest that these organisms release different types of vesicles capable of inducing an efficient immune response or modulate the immune response by either triggering [8] or inhibiting the production of pro-inflammatory cytokines [9]. Besides this immunomodulatory activity, EVs could serve as carriers for virulence factors [10]. The study of EVs from parasites includes helminths such as *Fasciola hepatica*, [11], *Heligmosomoides polygyrus* [12], *Schistosoma mansoni* [13] and *Echinostoma caproni* [14], as well as protozoa such as *Toxoplasma gondii* [15], *Trypanosoma cruzi* [16,17,18,19,20], *Leishmania* [21], different *Plasmodium* species [22,23] and anaerobic protozoan parasites such as *Entamoeba histolytica*, *Trichomonas vaginalis* and *Giardia intestinalis* [24], among others. More recently, and in the case of free-living amoebae with pathogenic potential, the secretion of EVs has been described in *Acanthamoeba*, a genus related to the production of fatal granulomatous amoebic encephalitis, keratitis and skin lesions. In this specific case, biological and nanomechanical properties of extracellular vesicles produced by clinical and environmental isolates of *Acanthamoeba* have been recently reported [25,26,27,28].

To our knowledge, the secretion of EVs by trophozoites of *Naegleria fowleri* (*N. fowleri*) has not been described. In this work, we confirm the secretion of EVs by a clinical isolate of *N. fowleri*, a thermophilic free-living amoeba that produces primary amoebic meningoencephalitis (PAM), an infection of rapid progression of the central nervous system that is fatal in more than 95% of cases [29]. Since the description of the first clinical case and until 2019, more than 440 cases have been reported worldwide [29]. However, for example, an underestimation of the disease of up to 50% has been proposed for the United States, which makes it difficult to know the true incidence of the infection [30].

During the pathogenic process of primary amoebic encephalitis, certain events may be relevant, such as the trophozoites’ ability to attach to the nasal mucosa, the chemotactic response to components of the nerves and the increased locomotion rate of the amoebae [31,32]. Moreover, a significant immune response through activation of the innate immune system [31], the phagocytic activity by structures known as food cups, and the release of cytolytic molecules (phospholipases, acid hydrolases, neuraminidases, pore-forming polypeptides such as naegleriapores and phospholipolytic enzymes) play a role in host cell and nerve destruction. This combination of both, pathogenic mechanisms of *N. fowleri* and the intense immune response resulting from its presence, produce significant nerve damage and subsequent central nervous system tissue damage [33]. However, some specific factors involved in the pathogenesis of PAM are still unknown and need extensive research. As an important step towards gaining a deeper knowledge on the production of EVs by *N. fowleri*, here we address the characterization of several physical and biochemical parameters, including the proteomic profiling of their cargo and the immunogenicity of some of their proteins. 

## 2. Materials and Methods

### 2.1. Axenic Culture of Naegleria fowleri Trophozoites

Trophozoites of a clinical isolate of *Naegleria fowleri* from Costa Rica (accession number: MT090627) [34] were cultured in 75 cm^2^ and 125 cm^2^ flasks (Nunc EasYFlask^®^, Thermo Fisher Scientific, Waltham, MA, USA) with 2% casein hydrolysate (Sigma Aldrich, St. Louis, MI, USA) culture medium, supplemented with 10% inactivated fetal bovine serum (Gibco, Grand Island, NY, USA) and antibiotics (penicillin/streptomycin). The flasks were incubated at 37 °C, with daily observation under an inverted microscope. The culture medium was replaced, at least, every two days. 

The manipulation of *N. fowleri* and products derived from this organism was performed in biosafety level 2 facilities.

### 2.2. Isolation of Extracellular Vesicles (EVs)

EVs of trophozoites of *N. fowleri* were obtained as previously described by Retana Moreira et al. with some modifications [19,20], following the Minimal Information for the Study of Extracellular Vesicles (MISEV) guidelines [35]. Briefly, amoebae were washed 3 times using sterile PBS and then, 5 × 10^7^ trophozoites were incubated for 5 h at 37 °C in 10 mL of 2% casein hydrolysate (Sigma Aldrich, St. Louis, MI, USA) culture medium without serum and antibiotics. After this incubation, the supernatants were collected and centrifuged at 2500× *g* for 15 min at 4 °C to discard possible remaining trophozoites. The resulting supernatants were collected for EV purification. 

In order to eliminate larger vesicles and obtain exosome-enriched pellets, supernatants were centrifuged at 17,000× *g* for 30 min at 4 °C and then filtered through 0.22 µm-pore membranes (Sartorius, Göttingen, Germany), for subsequent ultracentrifugation at 120,000× *g* for 150 min in a Sorvall™ WX80 Ultracentrifuge (Thermo Fisher Scientific, Waltham, MA, USA). The resulting pellets were washed twice in sterile-filtered (0.22 µm pore) PBS at 120,000× *g* for 150 min and finally resuspended in 150 µL PBS. The samples were analyzed immediately or stored at 4 °C for a maximum period of 4 days.

The viability of trophozoites after the 5 h secretion period was evaluated using the trypan blue exclusion test, and the protein concentration of each sample was quantified using the Micro-BCA protein assay kit (Thermo Fisher Scientific, Waltham, MA, USA). 

EV characterization techniques included transmission electron microscopy, atomic force microscopy, nanoparticle tracking analysis and dynamic light scattering, as previously reported [19,20] and briefly described below.

### 2.3. Transmission Electron Microscopy

To confirm the production of EVs by trophozoites of *N. fowleri*, pellets of the samples obtained ultracentrifugation were fixed in 500 µL of Karnovsky’s fixative (2.5% glutaraldehyde and 2% formaldehyde in 0.1 M cacodylate buffer; 50 mg of CaCl_2_ in 100 mL) for 2 h at 37 °C. After this step, 25 µL of the samples was placed onto a parafilm piece and a zinc grid with a carbon film was disposed over the samples for 5 min. After this time, two washing steps were performed using ultrapure water and the samples were negative-stained and contrasted with 1% (*v*/*v*) uranyl acetate for 1 min. The grids were dried with filter paper and the final examination was achieved under a Carl Zeiss LIBRA 120 PLUS SMT electron microscope (Carl Zeiss, Oberkochen, Germany). Three samples from different days of isolation were analyzed.

### 2.4. Atomic Force Microscopy

Topographic imaging of EVs obtained after the ultracentrifugation step was performed using the non-contact AFM mode in an NX-10 instrument (Park Systems, Suwon, Korea) with TAP300 cantilevers (K = 40 N m^−1^ and f = 300 kHz). Briefly, each sample was diluted 1:10 in sterile-filtered PBS and 8 µL of the dilution were deposited onto freshly cleaved muscovite mica. After 10 min, the substrate with the sample was carefully rinsed three times with ultrapure water (MilliQ^®^, Millipore, Burlington, MA, USA) to remove salts and finally air-dried. Images were typically acquired as 256 × 256 pixels at a scan rate of 0.3–0.5 Hz and further processed and analyzed using XEI software (Park Systems, Suwon, Korea). Representative images of each sample were obtained by scanning at least 3 different locations on at least 3 different samples.

To confirm the secretion of EVs by *N. fowleri*, trophozoites were washed 3 times by centrifugation at 800× g or 10 min using sterile PBS. Then, 5 × 10^4^ trophozoites were resuspended in 2% casein hydrolysate culture medium without serum and antibiotics and placed onto 18 mm round coverslips (Fisher Scientific, Portsmouth, NH, USA) that were previously rinsed and sterilized in an autoclave for 15 min at 121 °C. After 1 h of incubation at 37 °C, the coverslips with trophozoites were carefully washed in sterile filtered PBS and fixed in 4% paraformaldehyde for 30 min at room temperature. The coverslips were then washed three times in sterile filtered PBS (5 min each washing step) and air-dried, prior to atomic force microscopy analysis in a NX-10 instrument. For this purpose, the non-contact mode was also employed and images were acquired and analyzed as described.

### 2.5. Nanoparticle Tracking Analysis

Size, distribution and concentration of the EVs secreted by *N. fowleri* were determined by measuring the rate of Brownian motion according to the particle size in a Nanosight NS300 system (Malvern Panalytical, Worcestershire, UK). For the analysis, samples were diluted (1/100) in low-binding tubes (Eppendorf, Gamburg, Germany) using sterile-filtered PBS and measurements were performed at 25 °C. As previously reported, for data acquisition and information processing, the NTA software 3.2 Dev Build 3.2.16 was employed. The particle movement was analyzed by NTA software with the camera level at 16, slider shutter at 1200 and slider gain at 146, as previously reported [19]. The mean size distribution was calculated as a mean of three independent size distributions. 

### 2.6. Dynamic Light Scattering

Dynamic light scattering analyses of isolated EVs were performed using a Zetasizer nano ZS90 Size Analyzer (Malvern Panalytical, Worcestershire, UK), following sample preparation and measurement conditions as described for nanoparticle tracking analysis. The Zetasizer Ver. 7.11 software was employed for data acquisition and information processing and the mean size distribution was calculated as a mean of five independent size distributions (10 runs per measurement). 

### 2.7. Measurement of Zeta Potential 

Zeta potential of EVs was measured in a Zetasizer NanoZeta ZS90 system (Malvern Panalytical, Worcestershire, United Kingdom), at 25 °C. Briefly, EV samples were resuspended in 1 mL PBS, pH 7.4, and the electrophoretic mobility (μe) was determined by Laser Doppler electrophoresis. Zeta potential was calculated by using the Smoluchowski equation and results were obtained from at least four independent measurements (20 runs each).

### 2.8. Protein Pattern and Recognition of Extracellular Vesicles by Rat Polyclonal Anti-Naegleria fowleri Antibodies

#### 2.8.1. Preparation of Whole Protein Extracts of *Naegleria fowleri* Trophozoites

Whole protein extracts were obtained as follows: 5 × 10^7^ trophozoites of *N. fowleri* were washed three times in sterile PBS, resuspended in 500 µL PBS and submitted to sonication in a 4710 series ultrasonic homogenizer (Cole-Parmer Instrument, Vernon Hills, IL, USA) by applying 3 cycles of 30 s, with pause of 60 s between cycles. The resulting extracts were used immediately or stored in aliquots at −80 °C until use.

Protein quantification of the whole protein extracts was performed using the Micro-BCA protein assay kit (Thermo Fischer Scientific, Waltham, MA, USA). 

#### 2.8.2. Electrophoretic Separation of Proteins Using SDS-PAGE

To obtain protein profiles, samples of EVs and whole protein extracts of *N. fowleri* were diluted 1:1 in reducing sample buffer [36], heated for 10 min at 98 °C and subsequently loaded onto 12% SDS-polyacrylamide gels (SDS-PAGE). Electrophoretic runs were performed for 90 min (120 V) and proteins were finally visualized by Coomassie blue R-250 (BioRad, Hercules, CA, USA) and silver stains, following previously described protocols [37].

#### 2.8.3. Polyclonal Antibody Production

Two four-week old female Wistar rats were immunized with 40 μg of a whole protein extract of *N. fowleri* trophozoites (ATCC *N. fowleri* Carter 30808). The antigen was prepared by emulsification of the amoebae lysate (in sterile PSB) in complete Freund’s adjuvant (Sigma, Ronkonkoma, NY, USA), in a 1:1 ratio (final volume: 500 µL). This emulsion was administered intraperitoneally to the rats. For subsequent immunizations, the adjuvant was switched to incomplete Freund’s adjuvant (Sigma Aldrich, St. Louis, MI, USA). A total of 8 immunizations (1 per week) were performed. Finally, the animals were bled two weeks after the final immunization. The antibody response was evaluated using ELISA and Western blot, as described elsewhere [38].

#### 2.8.4. Western Blot

The recognition of proteins in EVs and trophozoites by rat polyclonal anti-*N. fowleri* antibodies was analyzed by Western blot. Briefly, SDS-PAGE was performed as described and proteins were transferred to nitrocellulose membranes (60 min, 90 V) in an Enduro VE10 Vertical Gel System (Labnet International, Edison, NJ, USA). Then, the membranes were blocked overnight with 5% non-fat milk in PBS-0.1% Tween 20, washed four times in a solution of PBS-0.1% Tween 20 and incubated for 1 h at room temperature with the polyclonal anti-*N. fowleri* serum (1:10,000). After the incubation, the membranes were washed and incubated for 1 h at room temperature with peroxidase-conjugated goat anti-rat IgGs (1:10,000) (Thermo Fisher Scientific, Waltham, MA, USA). After four washing steps with PBS-0.1% Tween 20, the fluorogenic substrate reaction was visualized using the Clarity ECL Western substrate (BioRad, California, United States) in a ChemiDoc imaging system (BioRad, California, United States).

### 2.9. Evaluation of Protease Activity of Extracellular Vesicles

In order to evaluate protease activity of EVs, zymographic assays were performed as described by Herron et al. [39]. Briefly, protein extracts of EVs and whole protein extracts of *N. fowleri* trophozoites were subjected to electrophoresis on SDS-polyacrylamide gels containing gelatin (1 mg/mL) [40]. Then, SDS was removed by washing the gels twice in 1% Triton X-100 (*w*/*v*) for 30 min, for subsequent incubation overnight at 37 °C in a developing buffer (50 mM Tris-HCl, 10 mM CaCl_2_, pH 8.0). After the incubation, gels were rinsed and finally stained with Coomassie brilliant blue R-250 (BioRad, California, United States). Areas of gelatin digestion, which indicate protease activity, were seen as non-stained regions. 

To characterize the general types of proteases present in EVs, the methodology described by Castro Artavia et al. [40] was employed. In this case, 1 mM phenylmethylsulfonyl fluoride (Sigma Aldrich, St. Louis, MI, USA) was employed as the serine proteases inhibitor, 1 mM 2-iodoacetamide (Merck, Hohebrunn, Germany) was selected as the cysteine proteases inhibitor and 10 mM EDTA (Sigma Aldrich, St. Louis, MI, USA) was employed as the metalloproteases inhibitor. To this end, EV samples were incubated for 30 min at 37 °C with each protease inhibitor and then submitted to SDS-PAGE in gels containing gelatin. Since EDTA is a reversible inhibitor, it was also included in the developing buffer.

### 2.10. Proteomic Analyses

#### 2.10.1. Protein Identification from SDS-PAGE/Western Blot Immunogenic Bands 

Immunogenic protein bands recognized by anti-*N. fowleri* antibodies in Western blots were matched to the corresponding bands on Coomassie blue stained gels. On the basis of immunoblotting results, 3 strongly recognized bands were manually excised from gels and submitted to tryptic digestion followed by nano-LC-MS/MS for protein identification, as described [41]. Proteins in gel plugs were reduced with 10 mM dithiothreitol, alkylated with 50 mM iodoacetamide and digested overnight in an automated workstation (Intavis, Tübingen, Germany) using sequencing-grade trypsin (Sigma Aldrich, St. Louis, MI, USA). The resulting peptides were dried, redissolved in water containing 0.1% formic acid and transferred to polypropylene nano-LC vials. Ten μL of each digest was loaded on a C_18_ trap column (75 μm × 2 cm, 3 μm particle; PepMap^®^, Thermo Fisher Scientific, Waltham, MA, USA), washed with 0.1% formic acid (solution A), and then separated at 200 nL/min with a 3 μm particle, 15 cm × 75 μm C_18_ Easy-spray^®^ analytical column using a nano-Easy^®^ 1200 chromatograph (Thermo Fisher Scientific, Waltham, MA, USA). A gradient from 0.1% formic acid (solution A) to 80% acetonitrile with 0.1% formic acid (solution B) was developed: 1–5% B in 1 min, 5–26% B in 25 min, 26–79% B in 4 min, 79–99% B in 1 min, and 99% B in 4 min, for a total time of 35 min. MS spectra were acquired in positive mode at 1.9 kV, with a capillary temperature of 200 °C, using 1 μscan at 400–1600 m/z, maximum injection time of 100 msec, AGC target of 3 × 10^6^, and orbitrap resolution of 70,000. The top 10 ions with 2–5 positive charges were fragmented with AGC target of 1 × 10^5^, maximum injection time of 110 msec, resolution 17,500, loop count 10, isolation window of 1.4 m/z, and a dynamic exclusion time of 5 s. The obtained MS/MS spectra were processed for peptide matching against protein sequences contained in the UniProt database for *Naegleria*, using Peaks X^®^ (Bioinformatics Solutions, Waterloo, ON, Canada). Cysteine carbamidomethylation was set as a fixed modification, while deamidation of asparagine or glutamine, and methionine oxidation were set as variable modifications, allowing up to 2 missed cleavages by trypsin. Parameters for match acceptance were set to FDR < 1%, −10 lgP protein score ≥ 20, and ≥1 unique peptides.

#### 2.10.2. Proteome Profiling of Whole EVs by Bottom-Up Shotgun Analysis

A preliminary proteome profiling of whole EV cargo was performed by a bottom-up shotgun strategy. To this end, EV proteins were concentrated by adding 4 volumes of cold acetone and incubating overnight at −20 °C [20]. The samples were submitted to centrifugation at 13,000× *g* for 10 min at 4 °C and two washing steps in 1 mL of cold acetone were performed. The acetone was then removed with a mixture of nitrogen and air stream and proteins were redissolved in one volume up to 12 µL of sterile PBS, mixed with electrophoresis sample buffer and loaded into wells of a 12% SDS-PAGE gel. The electrophoretic run was stopped as soon as the migration front entered 3 mm into the resolving gel, in order to concentrate the whole protein cargo as a single band in the stacking/resolving gel interface. This band was visualized by Coomassie stain, excised, destained in 50% acetonitrile, and finally submitted to tryptic digestion and nano-LC-MS/MS analysis as described above.

## 3. Results

### 3.1. Characterization of Extracellular Vesicles 

#### 3.1.1. Transmission Electron Microscopy and Atomic Force Microscopy

A standardized protocol that includes differential centrifugation, coupled to a filtration process through 0.22 µm pore membranes and ultracentrifugation was employed to evaluate the production of EVs by trophozoites of a clinical isolate of *N. fowleri*. After the isolation procedure, transmission electron microscopy and atomic force microscopy analyses were employed to identify the EVs secreted by this species.

In this work, 5 × 10^7^ trophozoites were placed in cell culture flasks of 125 cm^2^ with 10 mL 2% casein hydrolysate culture medium and incubated for 5 h at 37 °C to further isolate the EVs. Under our incubation conditions, amoebae produced approximately 0.675 µg/μL protein in EVs. After the 5 h incubation, viability of trophozoites remained above 97.5% (mean percentage of viability of amoebae remaining in the supernatant after the first centrifugation step at 2500× *g* and amoebae in the surface of the culture flask). 

**Figure 1:**
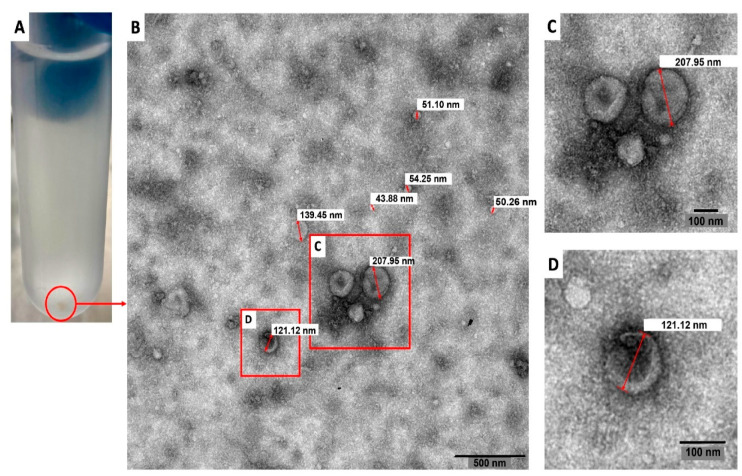
Transmission electron microscopy image of extracellular vesicles secreted by trophozoites of *Naegleria fowleri*. A pellet of extracellular vesicles obtained after the isolation procedure employed is shown in (**A**). The content of these pellets was analyzed using transmission electron microscopy and vesicles of different diameters were obtained (**B**) (43.88 nm; 50.26 nm; 51.10 nm, 54.25 nm; 121.12 nm; 139.45 nm and 207.95 nm in this image). Magnified images of (**C**,**D**) are also shown. Representative image of three different samples analyzed.

Figure 1 shows a representative transmission electron microscopy image of the pellets collected after applying the EV isolation protocol. In this Figure, diameters of the vesicles ranged from 43.88 nm to 207.95 nm. 

**Figure 2:**
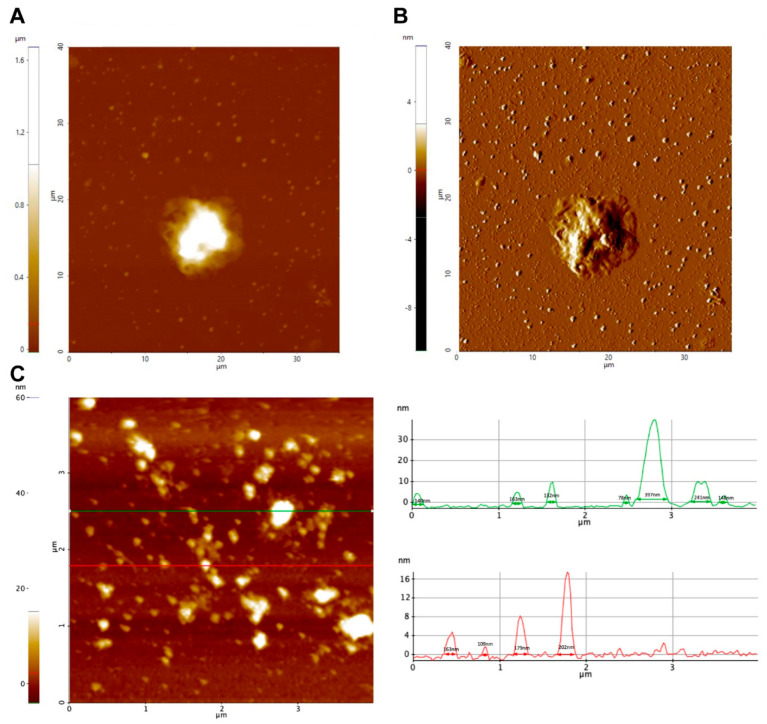
Atomic force microscopy analysis of extracellular vesicles of *Naegleria fowleri*. (**A**) Trophozoite surrounded by secreted extracellular vesicles (1 h incubation): (**A**) Z-height image and (**B**) amplitude image. (**C**) Analysis of isolated pellets of EVs revealed the production of vesicles ranging from 30 nm to 400 nm in length and 4.5 nm to 40 nm in height. Representative images are shown.

The secretion of EVs by trophozoites of *N. fowleri* was also analyzed using atomic force microscopy (Figure 2, Figures S1 and S2). In this case, pellets of EVs obtained after ultracentrifugation and trophozoites incubated for 1 h following the conditions for EV production were analyzed. Figure 2 shows topography (A) and amplitude (B) images of a trophozoite of *N. fowleri* surrounded by EVs of different sizes. A topography image of the extracellular vesicles obtained after the isolation procedure is shown in C. 

#### 3.1.2. Nanoparticle Tracking Analysis and Dynamic Light Scattering

**Figure 3:**
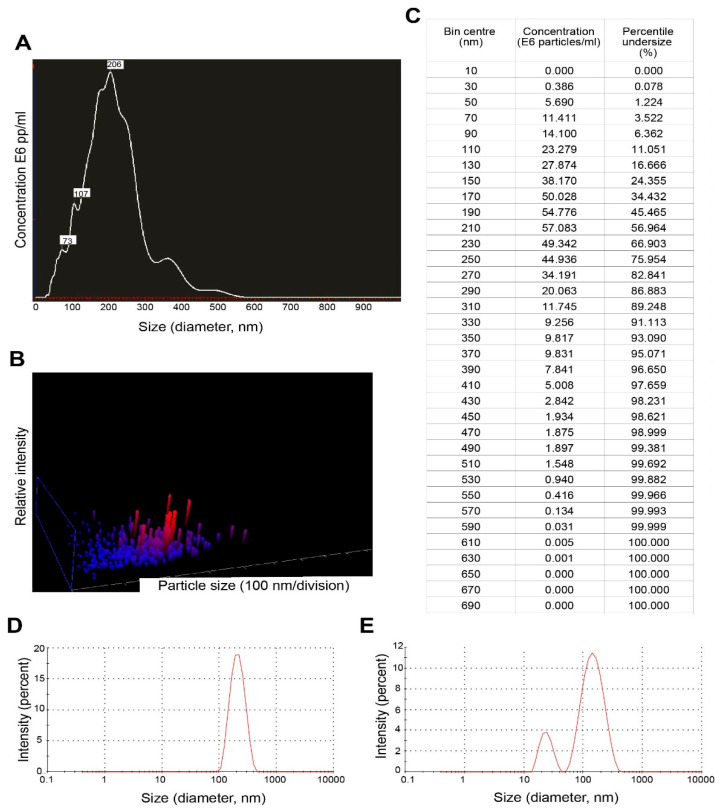
Nanoparticle tracking analysis and dynamic light scattering results of extracellular vesicles secreted by trophozoites of *Naegleria fowleri:* (**A**) Graph showing sizes and relative concentration of the vesicles using NTA; (**B**) 3D plot of particle size/relative intensity using NTA and (**C**) table of concentration of particles of different sizes obtained during the NTA analysis. (**D**) Graph showing the size of EVs from the pellets using DLS and (**E**) graph showing size of EVs from the remaining supernatant (after the washing steps) using DLS.

Results of nanoparticle tracking analysis (NTA) and dynamic light scattering are summarized in Figure 3. For EVs secreted by trophozoites of *N. fowleri*, NTA revealed a mean size of 216 ± 83 nm and a mode of 206 nm (A-C). Using the same technique, it was also possible to determine that 5 × 10^7^ trophozoites of *N. fowleri* secreted approximately 4.96 × 10^10^ particles. 

For dynamic light scattering (DLS), measurements of the pellets of EVs and 1 mL of the remaining supernatant at the bottom of the tube (collected after the washing step by ultracentrifugation) were performed (Figure 3D,E). For the pellets, the mean size obtained for EVs was 227.13 ± 37.98 nm, while the remaining supernatant contained vesicles of 206.29 ± 37.08 nm. A second population of EVs of 24.24 ± 9.18 nm was obtained in one of the replicates of the supernatant.

Finally, to characterize the surface electrical charge of EVs of *N. fowleri*, zeta potential measurements were performed in different samples, resulting in a mean value of −12.228 ± 4.843 mV.

### 3.2. Protein Profile and Recognition of Extracellular Vesicles by Anti-Naegleria fowleri Polyclonal Antibodies

**Figure 4:**
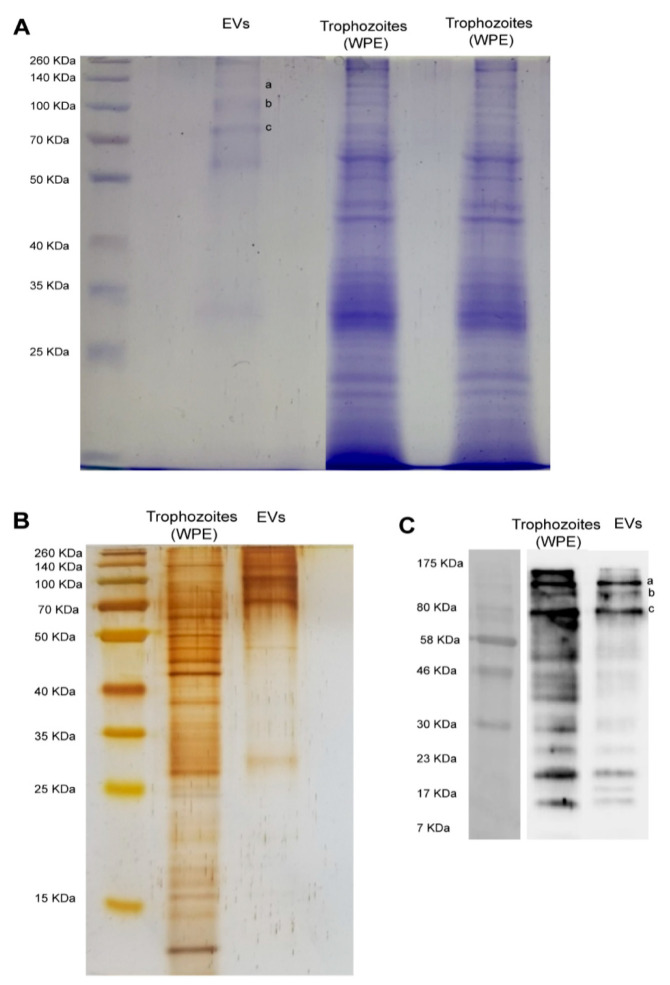
Electrophoretic (SDS-PAGE) protein profile of extracellular vesicles and protein recognition by polyclonal anti-*Naegleria fowleri* antibodies using Western blot. (**A**) Coomassie and (**B**) silver stains showing similar band patterns in extracellular vesicles, which range from >25 KDa to 260 KDa; EVs: extracellular vesicles; WPE: whole protein extracts of trophozoites of *N. fowleri*. For Coomassie stain, approximately 30 µg of protein/sample were loaded onto the gel and for silver stain, approximately 9 µg of protein/sample were loaded onto the gel. (**C**) Western blot showing the recognition of 3 intense bands over 70–80 KDa ((**C**), a–c) and 3 less intense bands under 23 KDa, using the polyclonal anti-*N. fowleri* antibody (1:10,000) produced in rat. Bands over 70–80 KDa ((**A**), a–c) were excised from Coomassie stained gels and submitted to nano-LC-MS/MS. Protein identifications are listed in Table 1.

Figure 4 shows the electrophoretic protein profile of EVs produced by trophozoites of *N. fowleri* after Coomassie and silver stains (A-B). Protein bands ranging from approximately 25 KDa to 260 KDa were evidenced. However, low molecular weight proteins seem to be less abundant in both stains. 

Western blot analysis using polyclonal anti-*N. fowleri* antibodies confirmed the immunorecognition of several EV proteins. Recognition was observed in bands from over 10 KDa to 175 KDa. The major recognition of proteins in EVs was observed close to 80 KDa, where 3 intense bands were identified. In addition, 3 less intense bands of <23 KDa were also observed (C). 

Preliminary whole proteome analysis of EVs revealed a total of 184 proteins (Supplemental Table S1), of which 85 are still uncharacterized in the current Uniprot database. The list of proteins includes Rho GTPases, actin and actin related proteins, ubiquitin, dehydrogenases and fowlerpain, among others.

### 3.3. Protease Activity of Extracellular Vesicles

**Figure 5:**
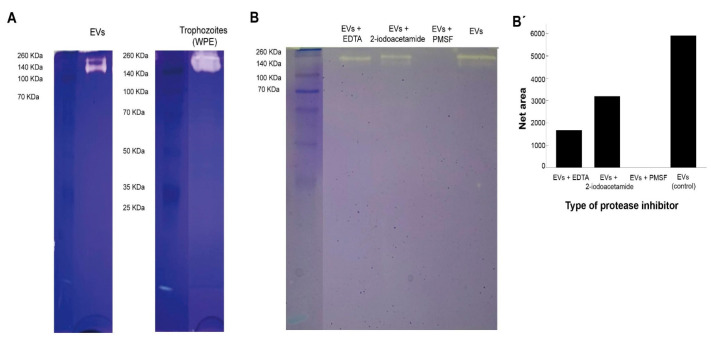
Protease activity of extracellular vesicles and whole protein extracts of trophozoites of *Naegleria fowleri* showing clear areas of gelatin digestion. For this experiments, 2.5 µg of protein/sample were loaded onto the gel. (**A**) Protease activity of extracellular vesicles (EVs) and whole protein extracts (WPE) of trophozoites of *N. fowleri*. (**B**) Specific protease activity of EVs previously incubated with EDTA 10 mM (metalloproteases inhibitor), 2-iodoacetamide 1 mM (cysteine proteases inhibitor) and phenylmethylsulfonyl fluoride (PMSF) 1 mM (serine proteases inhibitor). Protease activity was fully inhibited by PMSF, which could indicate a predominance of serine proteases in the samples analyzed. Densitometry analysis of the gel was preformed using Image J software and band intensities are represented in (**B′**).

Results from protease activity of EVs of *N. fowleri* are presented in Figure 5. EVs and whole trophozoite extracts were submitted to zymography using gels with gelatin. From the Figure, protease activity in both type of samples is observed (A). EVs and trophozoite extracts showed similar protease activity, which resulted more evident in whole trophozoite extracts. In both types of samples, proteases appeared in the region of high molecular weight (from approximately 100 KDa to 260 KDa). 

When the samples were treated with protease inhibitors, areas of gelatin digestion were not observed when incubating samples of EVs with phenylmethylsulfonyl fluoride, which could indicate the majority of proteases found in EVs of *N. fowleri* consist of serine proteases (B-B′). On the other hand, protease activity was partially inhibited when the samples were incubated with EDTA (metalloproteases inhibitor) and, apparently, less inhibition was found when incubating with 2-iodoacetamide (cysteine proteases inhibitor).

## 4. Discussion

EVs are small lipid vesicles released by prokaryotic and eukaryotic cells that contain nucleic acids, proteins, and small metabolites essential for cellular communication. Depending on the targeted cell, these vesicles can act either locally or in distant tissues in a paracrine, endocrine or juxtacrine cell signaling manner [42,43]. In EVs released by parasites, the analysis of the cargo and related functions suggest that these vesicles could be considered a mechanism of transferring biomolecules to host cells and tissues [44]. Recent reviews indicate that EVs released by protozoan parasites are capable of inducing an effective immune response, modulating the immune response and affecting the invasion process [45,46]. To our knowledge, this is the first study that evidences the production of EVs by *N. fowleri*; more specifically, a clinical isolate of this free-living amoeba that produces primary amoebic meningoencefalitis was employed.

In this study, trophozoites of *N. fowleri* incubated at 37 °C for 5 h in serum-free casein hydrolysate culture medium released EVs of different sizes. Transmission electron microscopy images showed vesicles ranging from ~44 nm to ~208 nm in diameter (Figure 1). Nanoparticle tracking analysis also revealed that ~76% of the vesicles were within <250 nm and only 2.3% of the population showed sizes of >390 nm (Figure 3), which could correspond to aggregates of vesicles, as ultracentrifugation could cause aggregation due to the high speed of centrifugation employed. The mean sizes of 206 nm or 227 nm, obtained by nanoparticle tracking analysis and dynamic light scattering, are slightly higher than those reported for EVs of *Acanthamoeba*, which range from 111.3 nm to 184.6 nm using DLS [27] and 166.7 nm using NTA [26]. For the latter genus, different size populations in the same sample of EVs were reported, especially when incubating trophozoites at 28 °C [27,46]. It is known that factors such as cell type, cell confluency or density, stimulation of cells with exogenous compounds, or abrupt change in culture conditions during the production of conditioned media might all potentially affect the yield outcome and the functional characteristics of the EVs secreted [47]. More studies are necessary to compare the EVs released under different culture conditions or by different isolates and/or species of *Naegleria*. 

Atomic force microscopy also confirmed the secretion of EVs by *N. fowleri* (Figure 2, Figures S1 and S2). In this case, samples of trophozoites and EVs collected after the isolation procedure employed were analyzed. For EVs, heights of 4.5 nm to 40 nm and diameters of 30 nm to 400 nm were obtained; diameters >220 nm could consist of EV aggregates. EVs of more heterogeneous sizes were observed when analyzing samples of trophozoites incubated at 37 °C for 1 h in serum-free casein hydrolysate culture medium. 

Besides imaging, particle number and size, determination of zeta potential was also included in the EV characterization analyses, which resulted in a value of −12.228 ± 4.843 mV. The zeta potential in a dispersed system is a measure of charge stability and affects particle-particle interaction [48]. This is considered a popular method to determine the surface potential of EVs, while used as an indicator of surface charge and colloidal stability, influenced by surface chemistry, bioconjugation and the theoretical model applied [49]. As the electrical charge of the surface of EVs is reflected in the zeta potential, it could be also considered as a characteristic of the vesicle´s population [19]. Determination of zeta potential is also considered one of the most useful tools to investigate the collective behavior of nanoparticles, such as EVs in dispersed systems, and thus, holds promise as a method for studying the activity of EVs in biological processes [49]. In this sense, the surface charge is known to influence different biological processes related to vesicles, such as cellular uptake and cytotoxicity. For example, charge-dependent differences in the mode of cytotoxic action have been reported using synthetic nanoparticles (NPs); it appears that positively charged NPs cause membrane damage, whereas anionic particles cause intracellular damage [50]. Studies on the effect of charge density and the kind of charge (positive, negative) in nonphagocytic cells have also shown that charged NPs (polystyrene and iron oxide) are taken up better than their uncharged counterparts [50]; moreover, anionic NPs seem to be better ingested in phagocytic cells [50]. These observations could suggest that, due to the mean zeta potential obtained in this study, EVs of *N. fowleri* could easily be captured by host cells and release their contents within the cells, including virulence factors, as will be discussed below. Uptake experiments to evaluate the entrance of EVs of *N. fowleri* in different cell lines are being currently performed by our research group. 

Literature reports that include zeta potential values of EVs of parasitic origin are very scarce. Mean values of zeta potential of −16 ± 4 mV and −18 ± 8 mV have been reported for trypomastigotes and epimastigotes of *Trypanosoma cruzi* [19]. In this study, the mean zeta potential value obtained in EVs secreted by trophozoites of *N. fowleri* is considered within the range previously reported by different EV research groups. It is important to mention that, according to several authors, there are still limitations when interpreting the results of zeta potential of EVs [51] and the isolation methodology should be taken into account when performing the interpretations.

To evaluate the presence of immunogenic cargo in EVs released by our clinical isolate of *N. fowleri*, Western blots were performed using polyclonal antibodies raised in rats against a whole protein extract of trophozoites of *N. fowleri* (ATCC *N. fowleri* Carter 30,808). Results showed a similar recognition pattern in EVs and protein extracts of trophozoites; however, in EVs, the stronger recognition was observed in a group of 3 bands above 70–80 KDa and 3 bands below 23 KDa (Figure 4). Similarities found in the recognition patterns of whole protein extracts and EVs suggest that antigenic components—either cytosolic or from the plasma membrane of trophozoites—could be packed within the EVs secreted by the amoeba, components that may possibly stimulate the immune response during the invasion process of the amoeba to the host. In recent years, the ability of EVs to elicit an effective immune response has been the subject of extensive research related to diagnosis and immunotherapy in parasitic diseases produced by helminths and protozoan microorganisms [8]. For example, in the case of malaria, evidence suggests that EVs are a source of circulating virulence factors that appear to influence disease severity [52]. Moreover, the release of EVs by *Plasmodium berghei* elicits a strong proinflammatory reaction thtrough macrophage activation [53], while the immunization with EVs of *P. yoelii* seems to induce a protective response in a murine model, triggering the production of IgG and stimulating reticulocitosys [22]. These findings suggest that EVs could be employed as antigen carriers for novel vaccination strategies. In this sense, it has been shown that EVs secreted by tachyzoites of *Toxoplasma gondii* are highly immunogenic, capable of inducing both protective humoral and cellular immune responses [54,55] and triggering the production of IL-10, TNFα and iNOS in an in vitro model using murine macrophages [56]. However, in another protozoan parasite, *Leishmania*, exosomes have shown an immunosuppresive role, inhibiting the production of IFN-γ and IL-10 by monocyte-derived dendritic cells [57]. Besides this immunomodulatory activity, EVs could act as vehicles for virulence factors like the major surface protease (MSP), also known as GP63 [58,59]. Regarding free-living amoebae, Costa et al. have demonstrated that EVs of clinical and environmental isolates of *Acanthamoeba* are capable of inducing the production of nitric oxide via TLR4 in an in vitro model using murine macrophages [28]. These authors concluded that EVs carry different types of PAMPs that could trigger the innate immune response during the infection process. 

In order to identify the predominant proteins in EVs recognized by the polyclonal anti-*N. fowleri* antibodies, the 3 more intense bands over 70–80 KDa were submitted to proteomic analysis. In this case, 36, 28 and 35 proteins were identified in bands a, b, and c, respectively. From these proteins, actin (B5M6J9), Hsp70 (Q6B3P1) and glyceraldehyde-3-phosphate dehydrogenase (D2W142) are proteins that had been previously reported by Gutiérrez Sanchez et al. in 2020, showing differential expression between *N. fowleri* and *N. lovaniensis* [60]. Another protein of interest is adenosyl homocysteinase (A0A6A5BYK8) (Table 1), a protein that has been considered as potential target in the design of new drugs for the treatment of primary amoebic meningoencephalitis. This protein participates in methylation reactions required for growth and gene regulation; for this reason, it has been considered an ideal target for antimicrobial drugs, especially for eukaryotic parasites [61]. Regarding peptidases, fowlerpain (a cysteine protease) and leucine aminopeptidase were also identified in EVs of *N. fowleri* (Table 1 and Table S1). In general terms, peptidases are considered virulence factors in different parasitic infections, being implicated in immune evasion and in host invasion. In *N. fowleri* infections, cysteine proteases present in trophozoites and conditioned media have been considered essential during the invasion process of the amoeba to the central nervous system and its survival in brain tissue [62,63]. It is important to highlight the presence of the elongation factor 1-alpha (eeEF1 α), identified in one of the bands (Table 1 and Table S1). This protein has been found in exosomes of *Leishmania* in early infections and has been identified as an important factor for immunosuppression and priming of host cells for *Leishmania* invasion [64,65]. 

Besides the identification of immunogenic proteins, a preliminary whole proteome analysis of *N. fowleri* EVs was also obtained by a shotgun approach, revealing the presence of 184 proteins in vesicles obtained under our experimental conditions (Table S1). The presence of Rho GTPases, actin and actin related proteins, ubiquitin, dehydrogenases and fowlerpain was confirmed in EVs (Table S1). However, 85 of the detected proteins are still uncharacterized in databases. Previous studies have identified the presence of 110, 148 and 130 proteins in EVs of another free-living amoeba, *Acanthamoeba*, grown under different experimental conditions [25,46]. Results obtained after quantitative analysis showed that the largest protein families in EVs of *Acanthamoeba* were categorized into the classes of hydrolases and oxidoreductases. 

Protease activity of EVs of *N. fowleri* was analyzed using zymography (Figure 5). In this case, protease activity between 140 KDa and 260 KDa, at 37 °C and pH 8, was identified. A complete and partial inhibition of protease activity was achieved when incubating EVs with phenylmethylsulfonyl fluoride and EDTA, respectively. These results suggest that the predominant enzymatic activity in EVs is related to serine proteases, followed by a less intense metalloprotease activity (Figure 4), similar to that described for clinical and environmental isolates of *Acanthamoeba* [25,26,28,46]. In contrast, published reports of protease activity of whole protein extracts of *N. fowleri* trophozoites suggest a predominance of cysteine proteases [63,66]. The release of serine proteases via EVs could contribute to the degradation of the extracellular matrix during the initial stages of the infection with this amoeba, thus facilitating tissue invasion as reported in in vitro infections with *Acanthamaoeba* genotype T4 [67,68,69]. Moreover, proteases could exert an effect over the immune response of the host, as reported for several parasites. For example, it has been demonstrated that serine proteases secreted by *Leishmania donovani* downregulate macrophages microbicidal activity [70], while in infections with *Acanthamoeba*, these enzymes are capable of inducing the production of IL-12 and mostly IL-6 by these cells [71].

In general terms, protease activity in protozoan related infections, including those produced by free-living amoebae, has been demonstrated and extensively discussed as a virulence factor. Proteases participate in the invasion and egress process of intracellular parasites like *Plasmodium falciparum* [72,73,74,75] and *Toxoplasma gondii* [76]; in the modulation of the immune response of macrophages during an infection with *Leishmania Mexicana* and *L. donovani* [77,78]; in the pathogenesis of the intestinal infection with *Entamoeba histolytica* [79,80], among others. Regarding free-living amoebae, the secretion and release of proteases has been related to the increase in cell permeability and in permeability of mitochondrial membranes, in extracellular matrix degradation, and in apoptosis induction and cell death [81,82]. More recently, the release in *Leishmania infantum* of the major surface protease, a metalloprotease responsible of the suppression of the immune response, was confirmed in EVs [59]. Ongoing studies to evaluate the significance of this secretion pathway of proteases in infections by free-living amoeba such as *N. fowleri* are being carried out in our laboratory.

## 5. Conclusions

Since 2012, scientific literature regarding EVs has increased at an exponential rate [83]. In parasitology, evidence suggests that EVs could be considered as key players in a pathway for the release of virulence factors. Research on EVs secreted by emerging pathogens, including free-living amoebae, could help to better understand pathogenic-related aspects in infections. The present study provides an extensive characterization of general properties and protein cargo for the EVs of a clinical isolate of *N. fowleri*, representing a valuable platform of information to pursue further investigations on this protozoon. Research in this field focusing on EVs could open new possibilities for the discovery of therapeutic targets and possible diagnostic biomarkers for infections such as the highly lethal primary acute meningoencephalitis caused by *N. fowleri*.

## Figures and Tables

**Table 1:** Proteins identified by nano-LC-MS/MS in trypsin-digested immunorecognized bands (a–c) of extracellular vesicles of *Naegleria fowleri* after SDS-PAGE/Western blot analysis using rat anti-*N. fowleri* antibodies *.

	Band a (~140 KDa)		Band b (~100 KDa)		Band c (~80 KDa)	
	Accession	Description	Accession	Description	Accession	Description
1	B5M6J9	Actin (fragment)	A0A6A5C9S6	Uncharacterized protein	A0A6A5BE22	Methylmalonate-semialdehyde dehydrogenase
2	A0A6A5BG15	Peptidase_S9 domain-containing protein	B5M6J9	Actin (fragment)	B5M6J9	Actin (fragment)
3	A0A6A5BDZ7	Uncharacterized protein	A0A6A5BQ42	Glucose-6-phosphate isomerase	A0A6A5BJW8	Uncharacterized protein
4	A0A6A5BS42	Uncharacterized protein	A0A6A5C1E4	Uncharacterized protein	A0A6A5C7K8	Cytosol_AP domain-containing protein
5	A0A6A5CCP5	Uncharacterized protein	A0A6A5C7K8	Cytosol AP domain-containing protein	A0A6A5BFY9	Peptidase_M28 domain-containing protein
6	A0A6A5CD16	VWFA domain-containing protein	A0A6A5C8E9	Guanine nucleotide-binding protein subunit β-like protein	A0A6A5BNQ4	Glutamate dehydrogenase
7	A0A6A5BUK4	Uncharacterized protein	A0A6A5BXF2	Histidine ammonia-lyase	A0A6A5BBK6	Uncharacterized protein
8	A0A6A5C3B7	Uncharacterized protein	A0A6A5CD16	VWFA domain-containing protein	A0A6A5CD16	VWFA domain-containing protein
9	A0A6A5BAB4	Uncharacterized protein	A0A6A5BR20	Uncharacterized protein	A0A6A5C9S6	Uncharacterized protein
10	A0A6A5BQ87	Uncharacterized protein	A0A6A5BHL2	CCT-alpha	A0A6A5BJJ6	Coronin
11	A0A6A5C150	C2 domain-containing protein	A0A6A5CCP5	Uncharacterized protein	A0A6A5C761	Uncharacterized protein
12	A0A6A5CG58	Uncharacterized protein	A0A6A5BT30	Uncharacterized protein	A0A6A5B5S1	ADP/ATP translocase
13	A0A6A5CHB1	Uncharacterized protein	A0A6A5C7Z0	Beta-hexosaminidase	A0A6A5CCP5	Uncharacterized protein
14	A0A6A5BVL0	Cytokinin dehydrogenase	A0A1L1XWF9	Fowlerpain-2	A0A1L1XWF9	Fowlerpain-2
15	A0A6A5BDH1	Prolyl endopeptidase	A0A6A5BP20	Pept_C1 domain-containing protein	A0A6A5BUU2	Bifunctional dihydrofolate reductase-thymidylate synthase
16	A0A6A5BXC9	Peptidase_S9 domain-containing protein	A0A6A5CCE5	SHOCT domain-containing protein	A0A6A5BUH9	Dihydrolipoyl dehydrogenase
17	A0A6A5BF81	Fructose-bisphosphate aldolase	A0A6A5C1F7	PRK domain-containing protein	A0A6A5BYK8	Adenosylhomocysteinase
18	D2W4E3	Leucine aminopeptidase **	D2V4W4	Mitochondrial chaperonin cpn60 **	A0A6A5BWP7	Catalase
19	A0A6A5BKE4	Alpha-mannosidase	A0A6A5C3B7	Uncharacterized protein	A0A6A5BF81	Fructose-bisphosphate aldolase
20	A0A6A5BP34	Uncharacterized protein	A0A6A5B5S1	ADP/ATP translocase	A0A6A5C539	WH2 domain-containing protein
21	A0A6A5C4U4	Peptidase_M3 domain-containing protein	A0A6A5C052	Uncharacterized protein	D2V5W7	Vacuolar proton pump subunit B
22	A0A6A5C7J0	Pyruvate carboxylase	A0A6A5C6S5	M20_dimer domain-containing protein	A0A6A5BFD6	UDP-glucose 6-dehydrogenase
23	A0A6A5C9S6	Uncharacterized protein	A0A6A5BBK6	Uncharacterized protein	A0A6A5BXJ0	AMP_N domain-containing protein
24	A0A1L1XWF9	Fowlerpain-2	A0A6A5BSJ3	Peptidase S53 domain-containing protein	A0A6A5BA58	Uncharacterized protein
25	D2VDR3	Methylcrotonyl-CoA carboxylase **	A0A6A5BFH5	Uncharacterized protein	A0A6A5CIW0	Non-specific serine/threonine protein kinase
26	A0A6A5C8W6	Uncharacterized protein	A0A6A5BUK4	Uncharacterized protein	A0A6A5BWG6	Uncharacterized protein
27	A0A4V8H039	Glyceraldehyde-3-phosphate dehydrogenase	Q6B3P1	Hsp70	A0A6A5BYB7	Uncharacterized protein
28	A0A6A5CIU9	Peptidase_S8 domain-containing protein	A0A6A5CFU9	Peptidase A1 domain-containing protein	D2V4W4	Mitochondrial chaperonin cpn60
29	A0A6A5CBE6	Uncharacterized protein			Q94626	Cpn-60 (fragment)
30	A0A6A5CHD7	Uncharacterized protein			A0A4V8H039	Glyceraldehyde-3-phosphate dehydrogenase
31	A0A6A5BGJ7	Imidazolonepropionate hydrolase			A0A6A5CAR5	Elongation factor 1-alpha
32	A0A6A5C721	RGS domain-containing protein			A0A6A5C052	Uncharacterized protein
33	Q25548	Penicillin amidase homolog (fragment)			A0A6A5BQM0	Isocitrate dehydrogenase
34	A0A6A5BBK6	Uncharacterized protein			A0A6A5C3B7	Uncharacterized protein
35	A0A6A5B2H1	Lysine--tRNA ligase			A0A6A5BUK8	Uncharacterized protein
36	A0A6A5BY41	Uncharacterized protein				

* Gel bands (a,b,c) of Figure 4 were excised, proteins were in-gel digested with trypsin and analyzed by nano-LC-MS/MS as described in Materials and Methods. ** Match with *Naegleria gruberi* entry in the Uniprot database for *Naegleria*. All other matches correspond to *N. fowleri*.

## Data Availability

Mass spectrometry raw files of the data presented in this work are available from the authors upon reasonable request.

## Supplementary Materials

The following supporting information can be downloaded at: https://www.mdpi.com/article/10.3390/biology11070983/s1. Figure S1: Atomic force microscopy images of a trophozoite of *Naegleria fowleri* surrounded by secreted extracellular vesicles: (A) Z-height image and (B) amplitude image; Figure S2: Atomic force microscopy images of extracellular vesicles secreted by trophozoites of *Naegleria fowleri*: A. and B. Z-height images, C. and D. amplitude images; Figure S3: Electrophoretic protein profile of extracellular vesicles and trophozoites of *Naegleria fowleri* after Coomassie stain; Figure S4: Electrophoretic protein profile of extracellular vesicles and trophozoites of *Naegleria fowleri* after silver stain; Figure S5: Molecular recognition of proteins of extracellular vesicles of *Naegleria fowleri* by polyclonal anti-*Naegleria fowleri* antibodies, using Western blot: A. molecular weight marker; B. samples of extracellular vesicles and trophozoites of *Naegleria fowleri;* Figure S6: Protease activity of extracellular vesicles (A) and trophozoites (B) of *Naegleria fowleri;* Figure S7: Determination of the protease types in extracellular vesicles of *Naegleria fowleri* using zymography. Table S1: Proteins identified in extracellular vesicles of *Naegleria fowleri* (total proteome).
