# Supplementary material for: Characterization of Extracellular Vesicles Secreted by a Clinical Isolate of Naegleria fowleri and Identification of Immunogenic Components within Their Protein Cargo
Source: Biology (Basel). 2022 Jun 29;11(7):983. doi: 10.3390/biology11070983 (PMC9312180; doi:10.3390/biology11070983)
Supplement: Supplementary file 1 [file biology-11-00983-s001.zip › Supplementary materials.pdf]

Supplementary materials

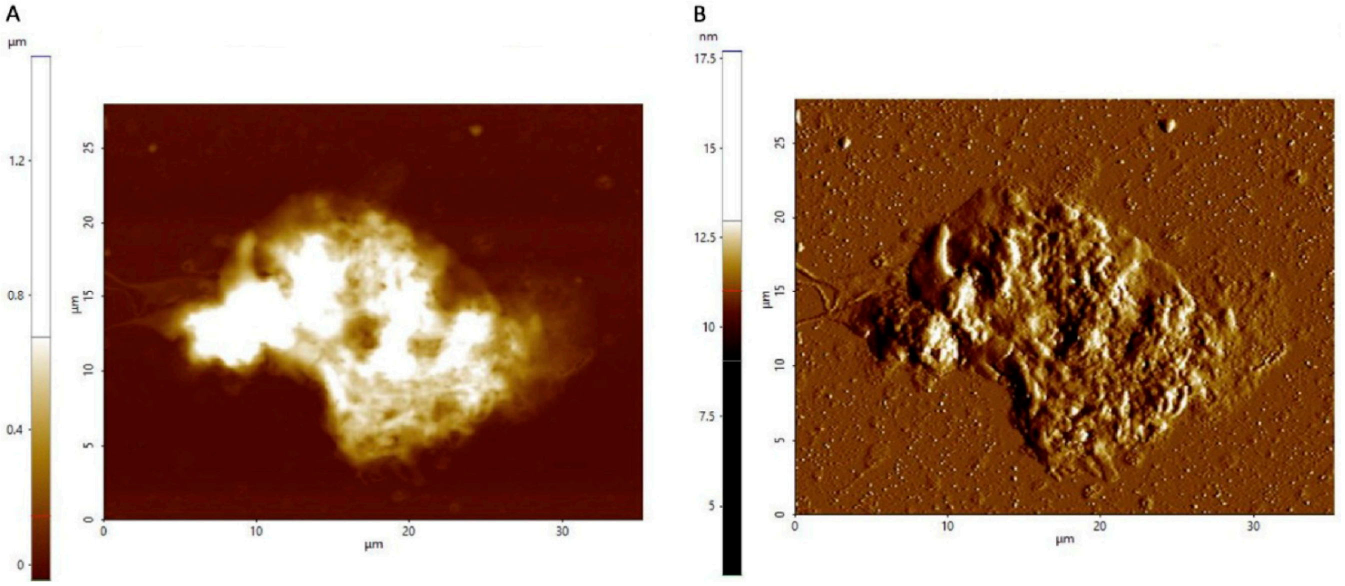

Figure S1. Atomic force microscopy images of a trophozoite of *Naegleria fowleri* surrounded by secreted extracellular vesicles: A) Z-height image and B) amplitude image.

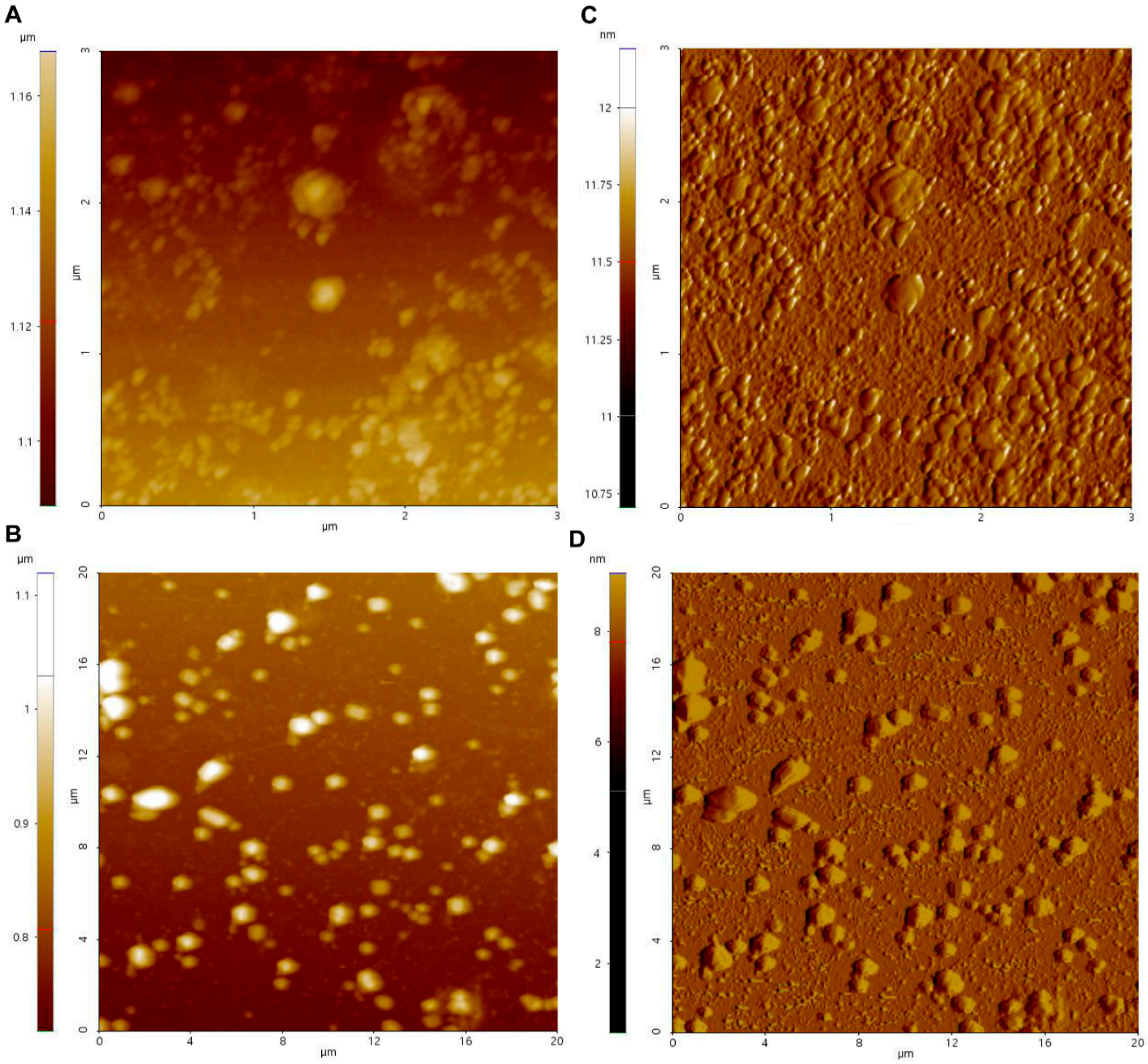

Figure S2. Atomic force microscopy images of extracellular vesicles secreted by trophozoites of *Naegleria fowleri*: A. and B. Z-height images, C. and D. amplitude images.

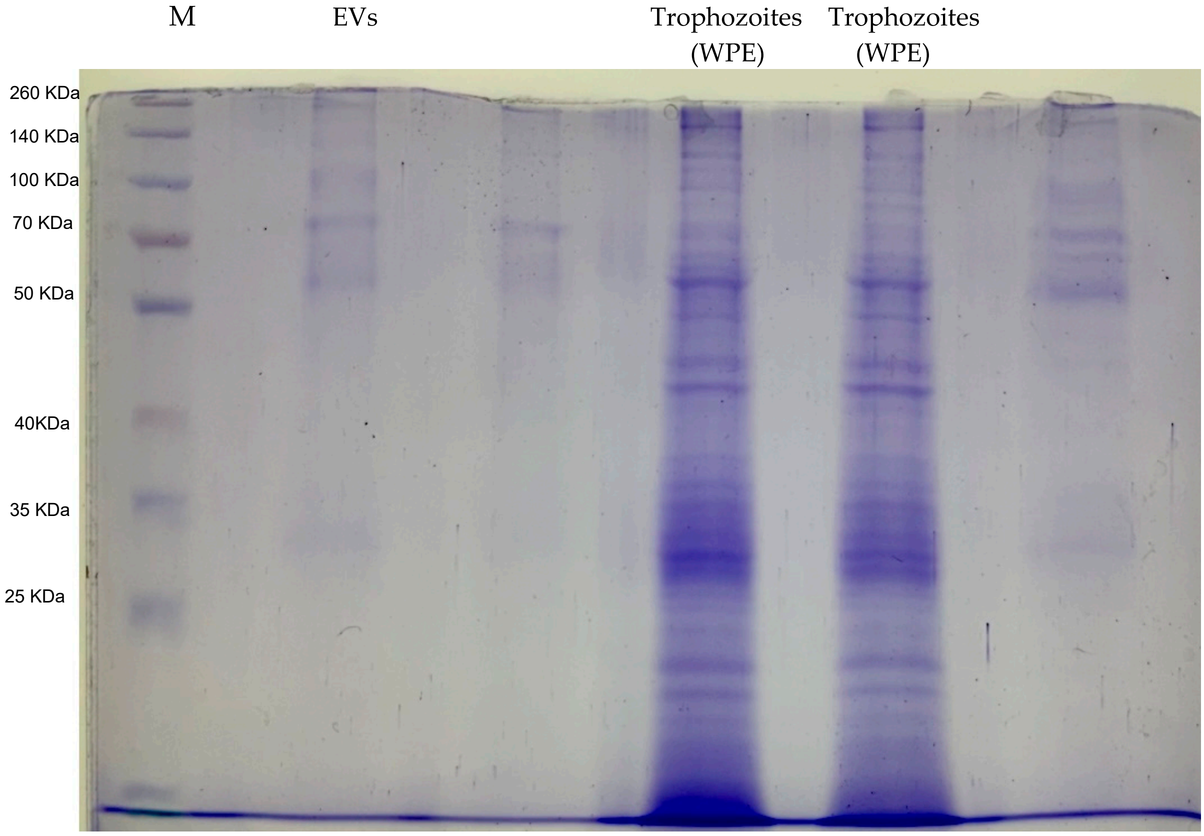

Figure S3. Electrophoretic protein profile of extracellular vesicles and trophozoites of *Naegleria fowleri* after Coomassie stain. M: molecular weight marker; EVs: extracellular vesicles of *Naegleria fowleri*; WPE: whole protein extracts of trophozoites of *Naegleria fowleri*.

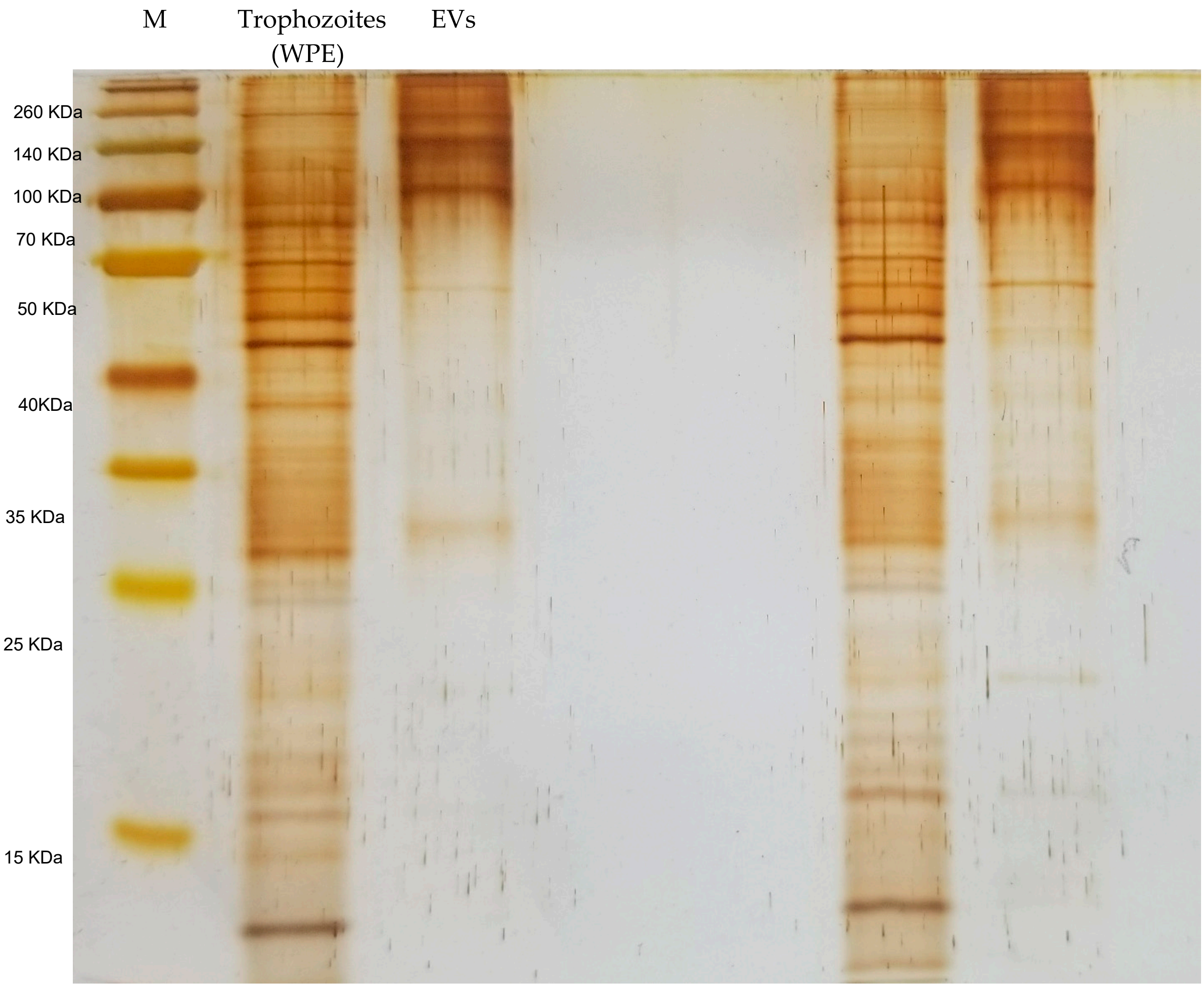

Figure S4. Electrophoretic protein profile of extracellular vesicles and trophozoites of *Naegleria fowleri* after silver stain. M: molecular weight marker; WPE: whole protein extracts of trophozoites of *Naegleria fowleri*; EVs: extracellular vesicles of *Naegleria fowleri*.

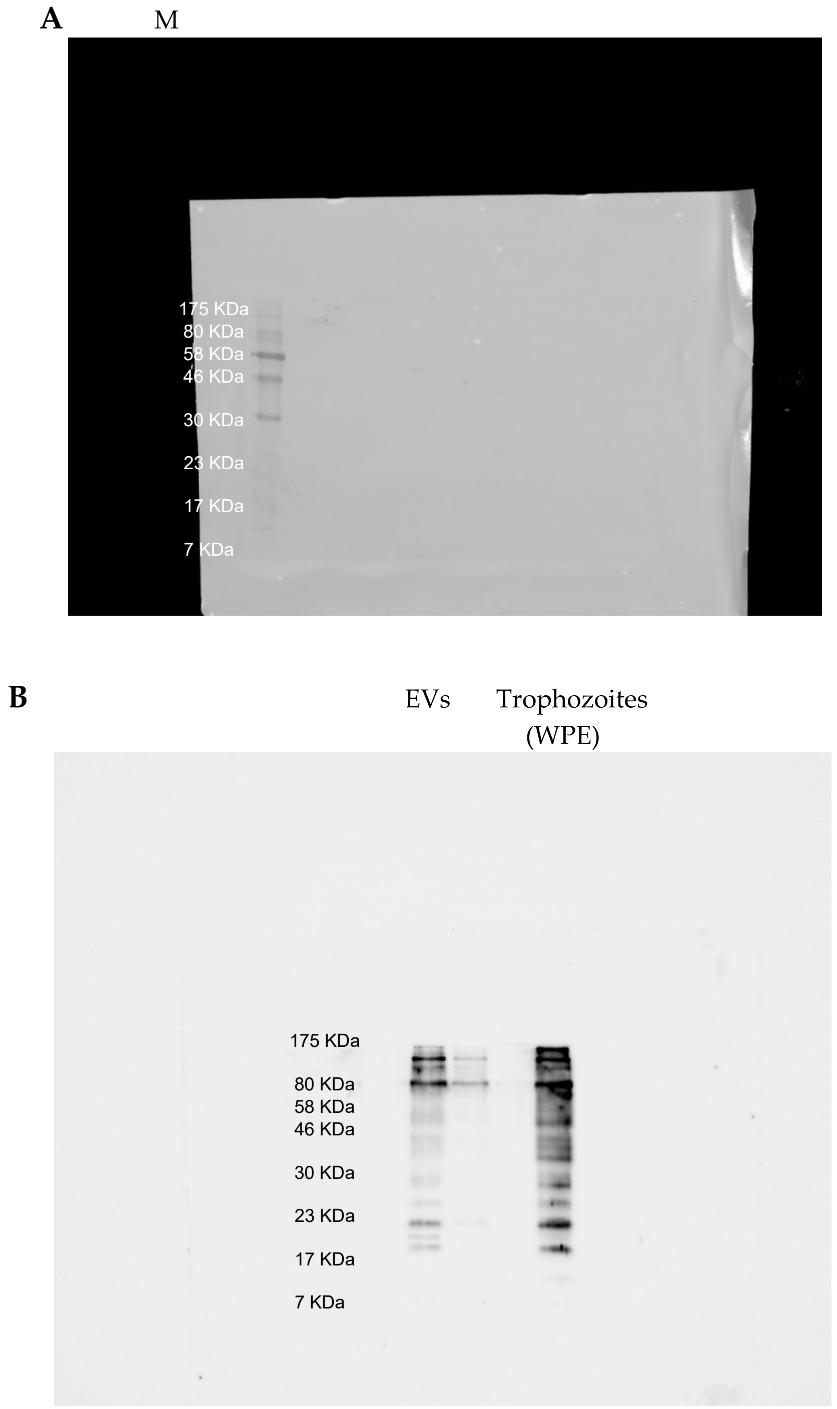

Figure S5. Molecular recognition of proteins of extracellular vesicles of *Naegleria fowleri* by polyclonal anti-*Naegleria fowleri* antibodies, using Western blot. A. molecular weight marker and B. samples of extracellular vesicles and trophozoites of *Naegleria fowleri*.

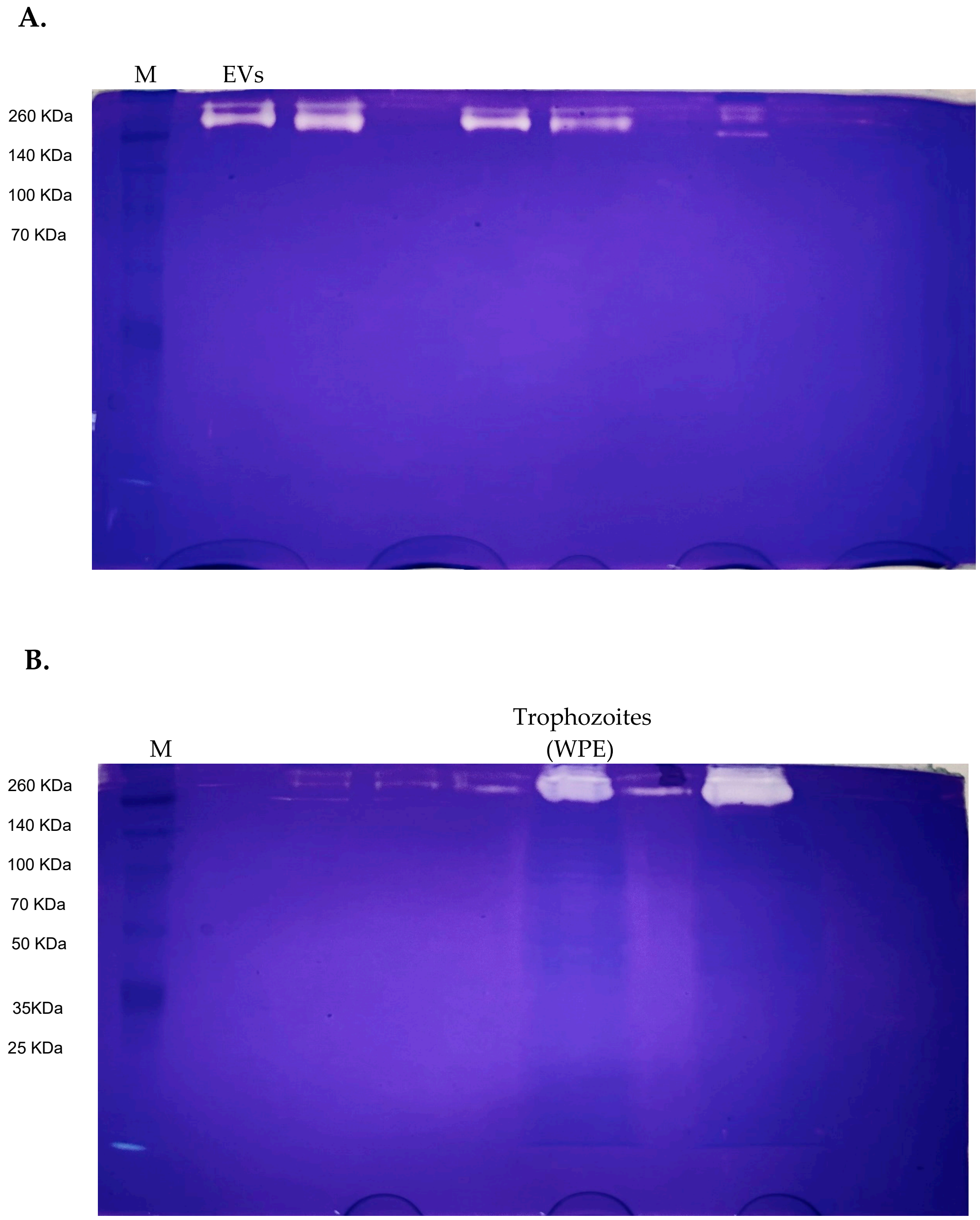

Figure S6. Protease activity of extracellular vesicles (A) and trophozoites (B) of *Naegleria fowleri.*

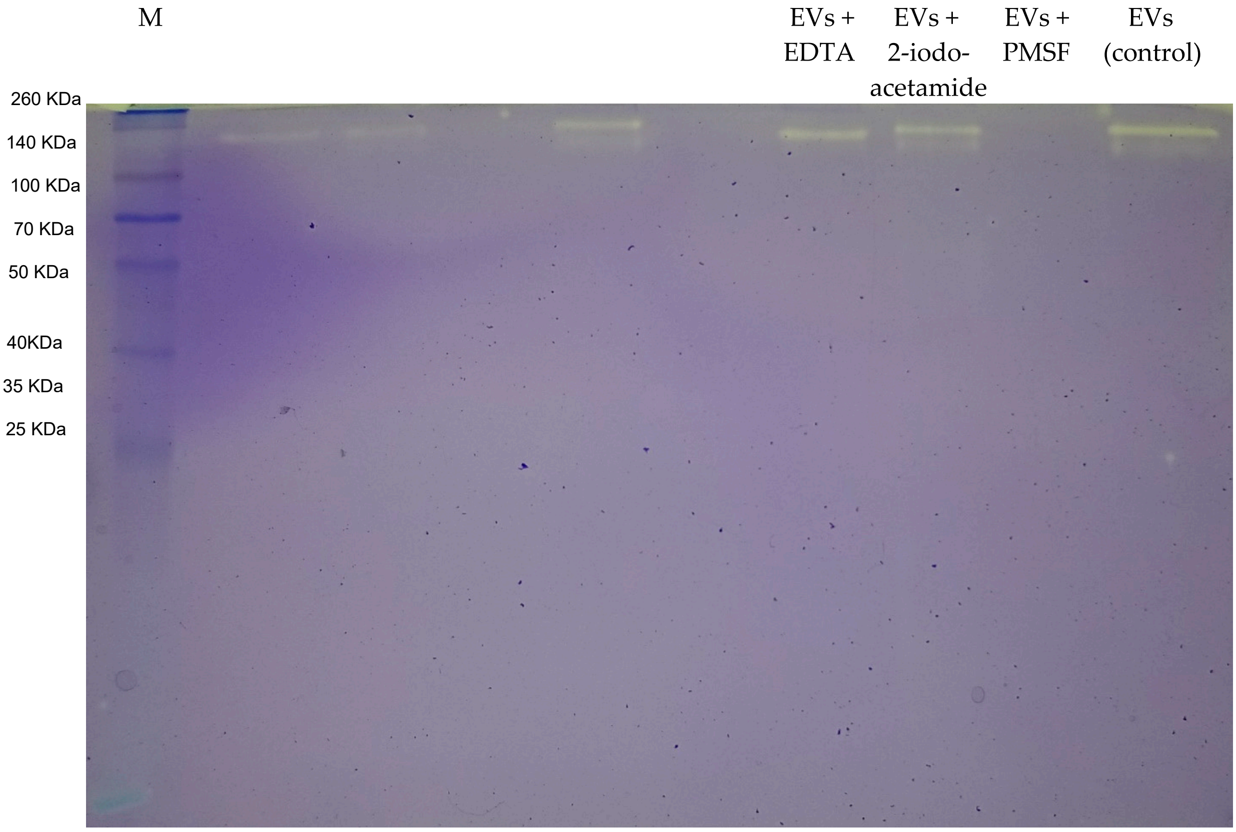

Figure S7. Determination of the protease types in extracellular vesicles of *Naegleria fowleri* using zymography. Samples of extracellular vesicles of *Naegleria fowleri* were incubated with specific protease inhibitors: EDTA 10 mM (metalloproteases inhibitor), 2-iodoacetamide 1 mM (cysteine proteases inhibitor) and phenylmethylsulfonyl fluoride (PMSF) 1 mM (serine proteases inhibitor). Extracellular vesicles without the incubation with inhibitors were employed as the protease activity control.
